# Evaluation of preventive care for swallowing difficulty through policy changes in Japanese long-term care insurance: analysis of a nationwide claims dataset for long-term care insurance

**DOI:** 10.1186/s12913-023-09990-8

**Published:** 2023-10-02

**Authors:** Hiroko Mori, Ayako Nakane, Haruka Tohara, Takeo Nakayama

**Affiliations:** 1 Section of Epidemiology, Shizuoka Graduate University of Public Health, 4-27-2 Aoi-ku, Kita-ando, Shizuoka-shi, Shizuoka, 420-0881 Japan; 2 Department of Health Informatics, Graduate School of Medicine & School of Public Health, Kyoto University, Yoshidakonoe-cho, Sakyou-ku, Kyoto, Kyoto 606-8501 Japan; 3 Division of Gerontology and Gerodontology, Department of Dysphagia Rehabilitation, Tokyo Medical and Dental University, 1-5-45, Yushima, Bunkyou-ku, Tokyo, 113-8510 Japan

**Keywords:** Long-term care facility, Dysphagia, Older adults, Policy evaluation, National claim database, Secondary analysis

## Abstract

**Background:**

Swallowing/feeding difficulty is a serious hidden health problem in the older population. Although oral intake services based on the degree of this difficulty have been provided and revised in Japanese public long-term care (LTC) insurance since 2006, their implementation has not been examined. We evaluated the impact of policy changes on these services.

**Methods:**

The nationwide database of LTC service uses in Japan was analysed for three oral intake services: Enhanced Oral Function in daycare facilities whose users were slightly disabled, Maintenance of Oral Ingestion and Transition to Oral Ingestion from tube feeding in residential facilities. Data were extracted for each June from 2015 to 2020 when monthly usage of these services was aligned and each June from 2009 to 2020 for the proportion of users according to LTC insurance certification. The major policy changes were the addition of municipal provision in Enhanced Oral Function and a requirement for multidisciplinary collaboration in Maintenance of Oral Ingestion in 2015. The impact of the medical fee reduction for developing percutaneous gastro-tubing to Transition to Oral Ingestion was also examined.

**Results:**

Between 2015 and 2020, the use of Enhanced Oral Function and Maintenance of Oral Ingestion increased and Transition to Oral Ingestion decreased, resulting in a total increase in use of 275,000 times or approximately 5,000 times per 100,000 among all older adults with LTC insurance certification. Concerning the proportion of users’ disability status, the major users of Enhanced Oral Function in 2020 were slightly disabled and independent older adults (70%, up from 55% to 2009). Regarding the major users of Maintenance of Oral Ingestion between 2013 and 2020, care-need level 5 (most severe) decreased by 11%, whereas the total of care-need levels 4 and 3 increased by 9%. The use of Transition to Oral Ingestion, which had been declining, showed a further decline after reduction of the medical fee for percutaneous gastro-tubing in 2014.

**Conclusions:**

Due to policy changes, Enhanced Oral Function and Maintenance of Oral Ingestion have increased in the number of use among slightly disabled persons. However, this increase may be insufficient given the hidden swallowing/feeding difficulty.

**Supplementary Information:**

The online version contains supplementary material available at 10.1186/s12913-023-09990-8.

## Background

Swallowing/feeding difficulty is a serious, progressive health problem in the older population, who are themselves rarely aware of it in the very early stages [1]. This difficulty has two aspects of both disease and disability. In the International Classification of Diseases version 11 (IDC-11), dysphagia is classified as difficulty in swallowing due to functional disorder (ICD-11 coding: MD93) and as functional swallowing disorder without structural abnormality (ICD-11 coding: DD90.1) [2]. In the International Classification of Functioning, Disability and Health, swallowing/feeding difficulty is related to all five of the following domains: environmental factors, individual factors, physical and mental structure, activity, and social participation [3]. Swallowing/feeding difficulty leads to malnutrition, respiratory tract infections and depression, hastens death [4], decreases the sense of well-being, causes a decline in social contact [5], and greatly affects the quality of life of family caregivers [6, 7]. The prevalence of dysphagia resulting from diverse causes, such as cerebral degenerative disease or cancer of the mouth, is easily identified. However, swallowing/feeding difficulty is a geriatric syndrome [8–10] that is reported to range from 13 to 53% among institutionalized older adults, depending on various criteria [11–15]. The care provided for swallowing/feeding difficulty is reported to be fragmented, without appropriate assessment of each person’s difficulties [16]. Hence, hidden swallowing/feeding difficulty can develop in older adults. If left untreated, the condition is directly related to malnutrition. Since eating practices enrich human interaction, early detection of hidden swallowing/feeding difficulty in the older population is essential for maintaining physical health and preventing social isolation. Furthermore, medical expenditures due to strictly diagnosed malnutrition in Japan were $14.5 billion, representing 4.3% of the national health expenditures in 2014 [17]. These health challenges and burdens on society are, of course, shared by other countries as well [18].

Since 2006 in Japan, long-term care (LTC) insurance has funded the following three oral intake services for older adults, based on the degree of swallowing/feeding difficulty: Enhanced Oral Function as comprehensive preventive care in daycare facilities, Maintenance of Oral Ingestion for older adults with clinically assessed dysphagia in LTC facilities, and Transition to Oral Ingestion from tube feeding in LTC facilities. The LTC insurance policy has been revised several times concerning these services regarding two main points. First, the provision of Enhanced Oral Function by municipalities in addition to basic prefectural services was expanded to strengthen the preventive services of LTC insurance. Second, Maintenance of Oral Ingestion has required dietary observation and collaborative meetings involving multiple health professions to improve the service content to better suit the person.

A multidisciplinary approach is considered important in care for these patients because swallowing/feeding function involves multidimensional factors such as the physical function of multiple organs, shape of the food mass, skills in dietary care, and body position during meals [16, 19, 20]. Previous studies have reported the impact of educational interventions of Enhanced Oral Function [21] and changes in the income of LTC facilities due to the participation of dentists [22]; however, little is known about the effect of these policy changes in the LTC insurance system or their impact on LTC services. This study aimed to describe the impact of policy changes on the number of services used and evaluate users’ differing disabilities by mining the Nationwide Open Database of LTC Service Uses in Japan.

## Methods

### Japan’s public LTC insurance

Japan’s public LTC insurance system has taken on the responsibility of caring for older adults as a whole society since the system’s introduction in 2000. Three administrative bodies perform distinct roles in Japan’s public LTC insurance scheme (2000): the national government sets policies, the prefectures designate and supervise facilities and providers of LTC insurance services on a basic level, and the municipalities are responsible for the actual provision of services. When an older adult (aged > 65 years with LTC insurance approval, hereafter “the user”) in the community applies to use LTC services, the municipality first inspects his or her physical and mental health to assess the individual’s eligibility for the services [23]. A care plan is then formulated according to the recommendations of the specialist and the wishes of the user. The LTC insurance policy establishes a limit on the availability of services based on the severity of LTC insurance certification.

### Statistics from the ministry of health, labour and welfare

Data for Enhanced Oral Function, Maintenance of Oral Ingestion, and Transition to Oral Ingestion services were obtained from the Nationwide Open Database of LTC Service Uses in Japan. This administrative database contains the monthly usage of each service measured by all LTC insurance claims since 2009, as well as the frequency of use of each service according to the LTC insurance certification care-need level. However, it does not include information on the users’ sex or age. The data are publicly available free of charge via the internet as accumulated data on LTC insurance services throughout Japan. As policy changes were enacted in April, which is the beginning of the fiscal year in Japan, we used annual data for the month of June. The number of new percutaneous endoscopic gastrostomy (PEG) and percutaneous transoesophageal gastrotubing (PTEG) procedures (excluding PEG for drug administration from 2018) were extracted from the National Medical Fee Database for June annually between 2009 and 2020. After noticing changes in service offerings and the consolidation or reorganization of LTC residential facilities within the study period, these data were also extracted. The number of qualified users of each service was calculated using the eligible daycare user and LTC facility user data, and the number of uses by these qualified users was calculated. This study followed the reporting guidelines of the STrengthening the Reporting of OBservational studies in Epidemiology (STROBE) Statement and the REporting of studies Conducted using Observational Routinely collected Data (RECORD) Statement [24].

### Policy changes in LTC insurance

We focused on two aspects of LTC insurance that are revised every three years: expansion of the providing system and revision of the service content. We also analysed the impact on the LTC insurance service of a policy change to the Medical Fee. LTC insurance is revised every three years, and the Medical Fee is revised every two years. Important changes due to these revisions are as follows. First, the scope of LTC insurance was expanded to cover a new preventive care service system in 2015, and municipalities were given the authority to provide this new component of the system. Second, the Maintenance of Oral Ingestion changed in 2012 with the approval of dentists to participate and in 2015 with the requirements for multidisciplinary collaboration and daily dietary observation. Third, the fee for new PEG was reduced in the 2014 amendment of the Medical Fee policy, and the requirement criteria for medical facilities to perform them were tightened. This change may have affected the number of older adults eligible for Transition to Oral Ingestion.

### LTC support services for swallowing/feeding difficulty

#### Enhanced oral function

This service provides comprehensive oral management, including oral hygiene and salivary gland stimulation, in LTC facilities such as daycare, rehabilitation, daily life support services, and dementia-friendly daily life care (Table 1).

**Table 1:** Summary of LTC* insurance services for swallowing/feeding difficulty

LTC insurance service		Contents of service		Setting		Feeding status of targeted older adults		Outline of requirement for benefit claims
Enhanced Oral Function		Comprehensive oral care including oral hygiene		Daycare facility		Unaware of oral intake disability		Prevention service of LTC. For older adults who have problems with swallowing, eating, or oral hygiene. Facility requirements include at least one speech-language-hearing therapist, dental hygienist, or nursing staff.
Maintenance of Oral Ingestion: type 1 in 2015†		Specializing in oral intake care		Residential facility		Clinically determined dysphagia		After planning by physicians and dentists based on clinical swallowing assessment, multidisciplinary conferences are conducted (dietitians, nurses, nursing support staff) and dietary observation is performed. Nutrition Management Service practices are a prerequisite for this service addition. Before 2015, service provision was on a daily basis and there was no specific regulation on content. Service provision has been on a monthly basis since 2015.
Transition to Oral Ingestion		Oral care with special emphasis on oral recovery for tube-fed persons		Residential facility		Nutritional intake by tube feeding		Services specific to tube feeders. Nutrition Management Service practices are a prerequisite for this service addition. This service can only be practised once per resident and the duration is 180 days. Even if the period of service is longer than 180 days, the period can be slightly extended with a physician’s order.

*LTC, long-term care

† Policy change in 2015 established two types of Maintenance of Oral Ingestion Type 1: services received in a residential facility, and Type 2: additional to Type 1 if the residential facility is linked to a specific dental clinic. Hence, type 2 was excluded from the data in this study because this study was an investigation of the number of times of use

The regulations for this service do not specifically require management of the nutritional status of older adults. The status of swallowing/feeding difficulty is assessed using a checklist in parallel with xerostomia and denture status by nonmedical staff. The service is positioned as a primary preventive measure, i.e., a measure to detect and prevent the onset of dysphagia in older adults who are not aware of a latent oral problem. This service provides oral cleaning, dental care, and oral function training without a set program [25]. Facility staff, dental hygienists, and speech-language-hearing therapists are assigned to the daycare facility to provide this service, but no dentist or physician is required to participate.

#### Maintenance of oral ingestion

This service addresses dysphagia in older adults by clinical evaluation in residential LTC facilities as a secondary preventive service, with the requirement for continuous monitoring of the nutritional status of each resident by a registered dietitian (Table 1). This monitoring is an independent LTC insurance service known as a Nutrition Management Service. Facilities that can provide this service include nursing homes as a place to live for those with LTC insurance care-need levels ≥ 3, geriatric healthcare facilities as a transition in returning home from a medical institution, sanatorium medical facilities, and hybrid facilities for medical care and LTC. Small-scale residential facilities specializing in dementia do not provide these services. The service includes a three-month care plan that is tailored to the individual’s feeding condition. This service was amended in 2012 and 2015. In the first revision, dentists, in addition to physicians, were approved to participate in the development of oral care plans. Technical guidance and education for facility staff were left to the discretion of individual physicians and dentists. In the 2015 revision, multidisciplinary collaboration meetings involving physicians, dentists, registered dietitians, nurses, speech-language-hearing therapists, dental hygienists, and care support specialists became mandatory in addition to dietary observation. Furthermore, the basis of service provision was changed from daily to monthly (Table 1). Specifically, this policy change established two types of service: type 1 for services received at a residential facility and type 2 for an addition to LTC insurance for residential facilities affiliated with a specific dental clinic. As this study examined the number of times that a service was used, only type 1 information was included in the present analysis.

#### Transition to oral ingestion

This service is provided to support the shift to oral intake in older adults who receive tube feeding in residential LTC facilities (Table 1). The service is limited to a one-time use per older person for a basic period of 180 days and requires Nutrition Management Service by a registered dietitian under the physician’s direction as well as collaboration with a speech-language-hearing therapist or a member of nursing staff. Eligible facilities are the same as those for Maintenance of Oral Ingestion.

## Results

**Fig. 1:**
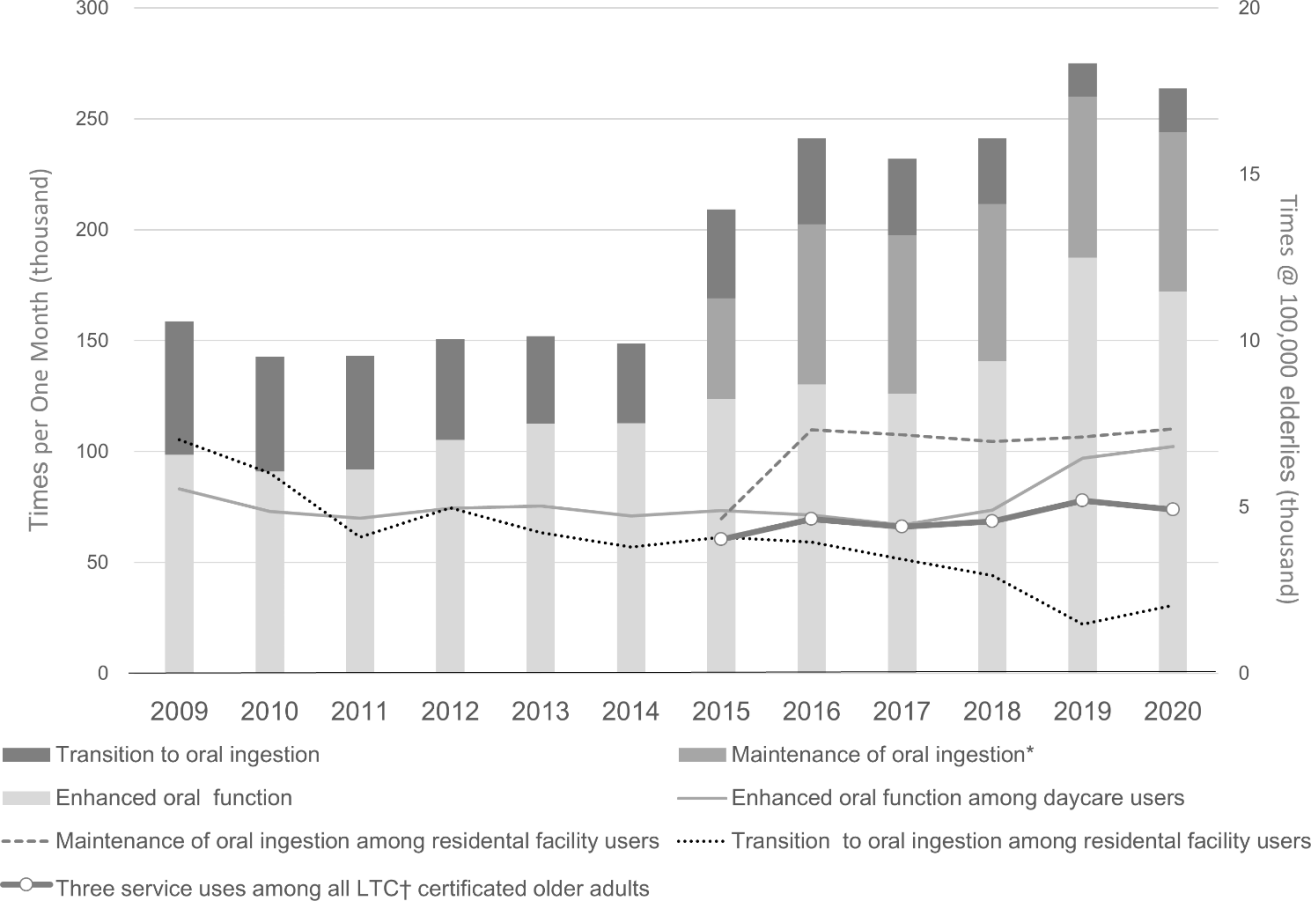
Monthly use and calculated use (per 100,000 older adults) of each oral related service * Prior to 2015, this service was provided on a daily basis. Therefore, services provided between 2009 and 2014 were excluded from the study ^†^LTC, long-term care

The calculated number of monthly uses for any of the three services per 100,000 persons certified for all LTC insurance increased from 4,000 to 2015 to approximately 5,000 in 2020 (Fig. 1). The transition for each service was shown in terms of the number of uses per 100,000 persons for whom that service is available. The real number of uses of Enhanced Oral Function in daycare facilities increased 1.8-fold from 98,500 times in 2009 to 172,200 in 2020, whereas the number of uses per 100,000 daycare users increased 1.2-fold from 5,500 to 6,800 times. Regarding Maintenance of Oral Ingestion, data from 2015 after the revision to reporting on a monthly basis showed an increase in the real number of users from 45,400 to 2015 to 72,200 in 2016, and this rate stabilized at approximately 70,000 from 2017 onwards. Use per 100,000 residential facility users stabilized in 2016 at approximately 7,000 per month. The real number of uses of Transition to Oral Ingestion showed a gradual decrease from 2016 onwards and was 15,000 in 2019.

**Fig. 2:**
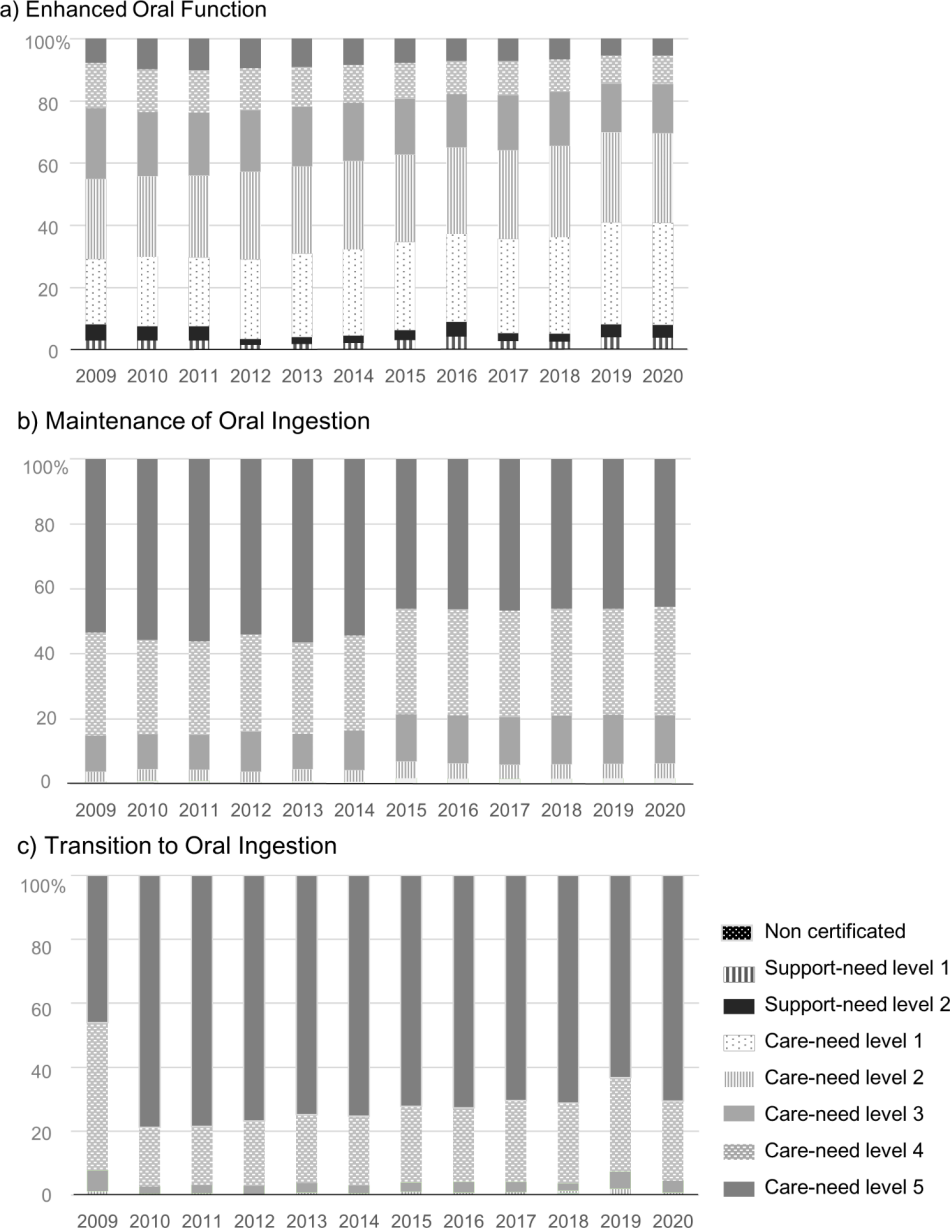
Use of each oral intake service according to the certification status of LTC* insurance *LTC, long-term care

**Fig. 3:**
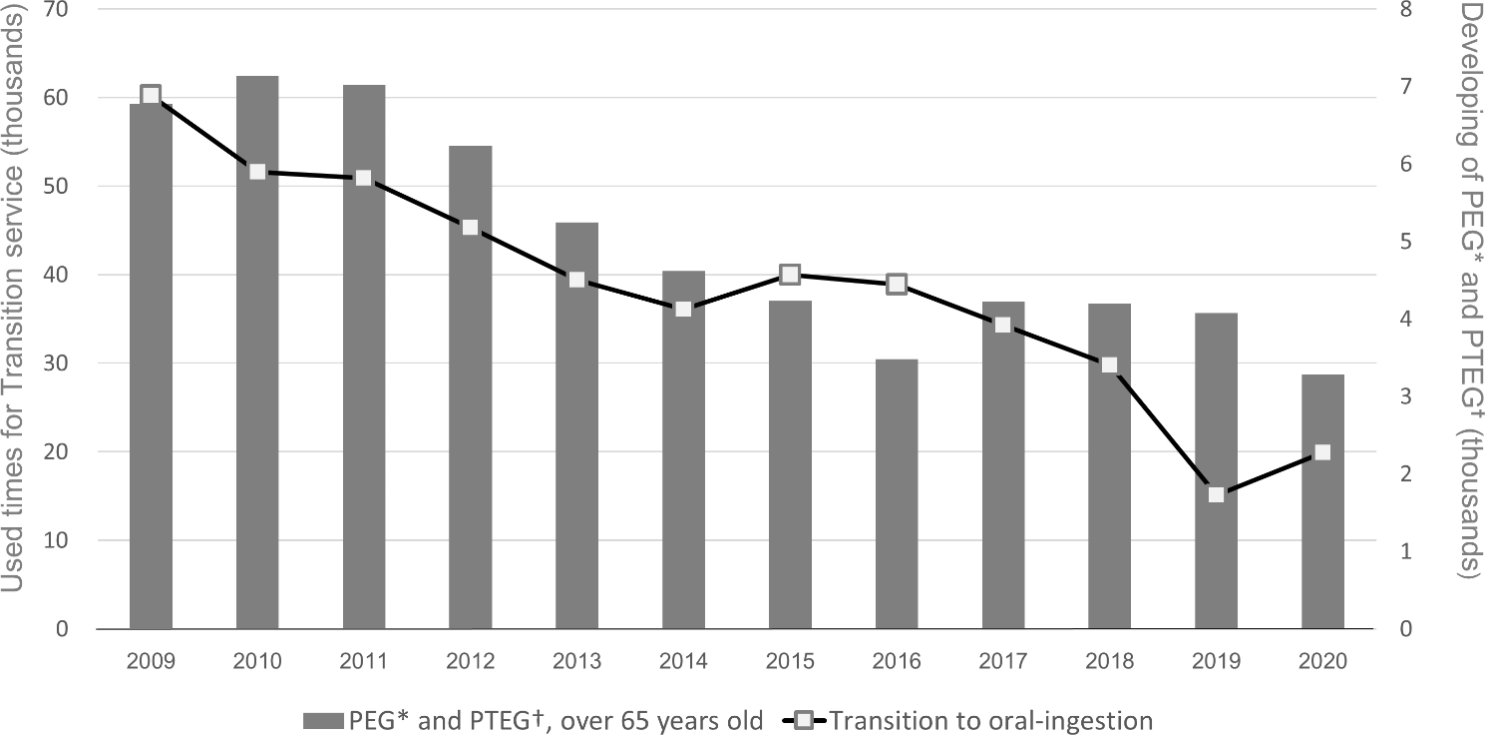
Trends in new developments of tube feeding and use of the Transition to Oral Ingestion Service. *PEG, percutaneous endoscopic gastrostomy. †PTEG, percutaneous transoesophageal gastro-tubing

Figure 2 shows the proportion of use of each service according to the user’s LTC insurance certification status. The policy change in LTC insurance began providing services to older adults without LTC insurance certification in 2015. Of the three services covered, Enhanced Oral Function was primarily affected by this policy change. However, uncertificated older adults accounted for less than 1% of service users. The proportion of older adults with light disabilities (certified as LTC insurance care-need levels 1–2) and independent older adults (uncertified and those with LTC insurance support-need levels 1–2) receiving this service gradually increased from more than 55% in 2009 to 70% in 2020. However, the proportion of independent older adults was relatively unchanged, from 8.2% to 2009 to 8.6% in 2020. Maintenance of Oral Ingestion as secondary prevention remained unchanged at approximately 95% for older adults with severe disabilities (certified as LTC insurance care-need levels 3–5). However, a closer look at the proportions of users according to care-need level revealed an 11% decrease in use by those with care-need level 5 and a 9% increase in use by the combined number of those with care-need levels 3 and 4 in 2020 compared with 2013, when use by those with care-need level 5 was the highest throughout the analysed period. Concerning Transition to Oral Ingestion in residential facilities for those receiving tube feeding, older adults with severe disabilities consistently accounted for nearly 98% of users between 2009 and 2020. Figure 3 shows the trends in new PEG and PTEG and in the use of Transition to Oral Ingestion. Use of this service was declining even before the Medical Fee revision for new PEG in 2014 (Fig. 3).

**Fig. 4:**
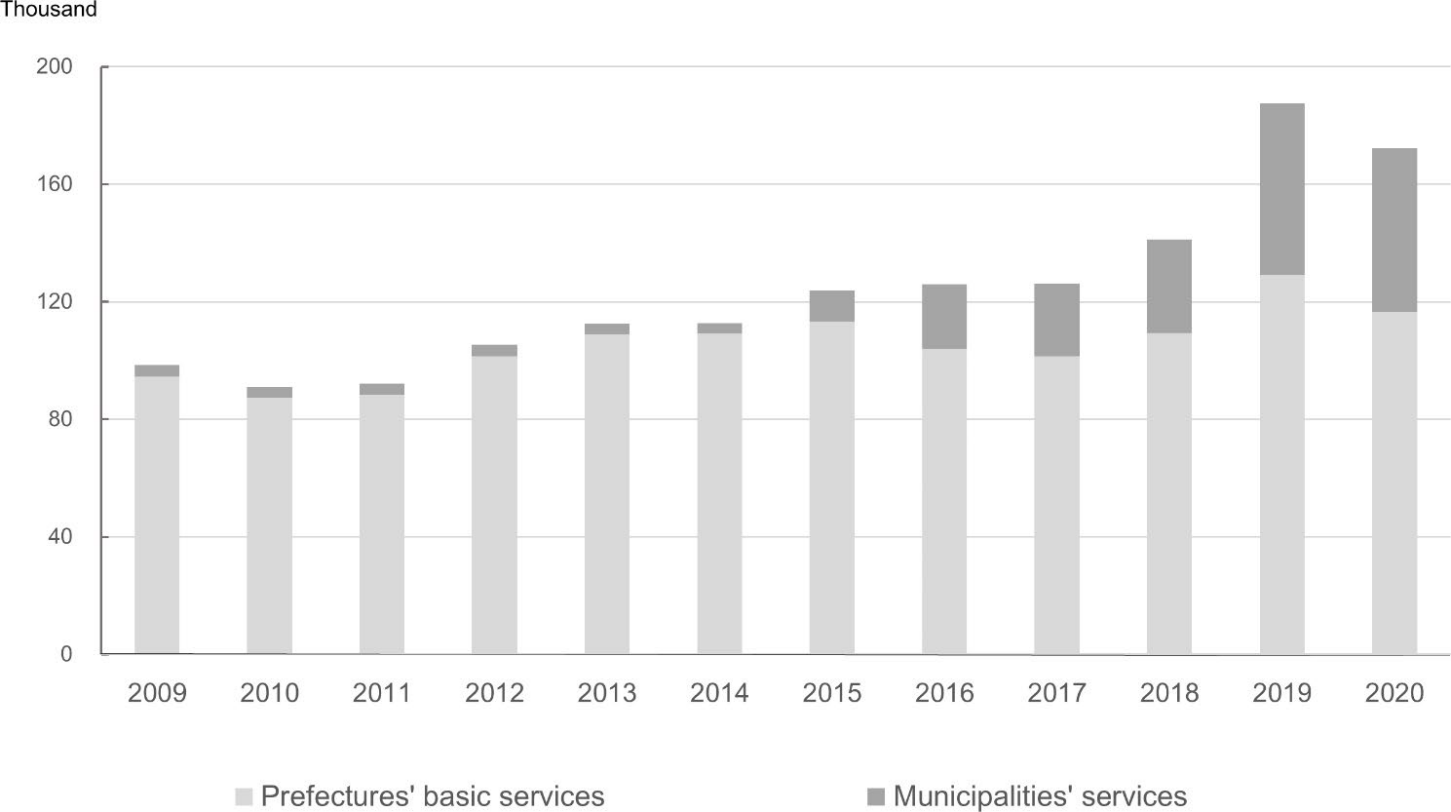
Use of Enhanced Oral Function services by prefectures and municipalities

Figure 4 shows the increase in the use of Enhanced Oral Function driven by the provision of the service at the municipal level since 2015. The number of LTC services provided under supervision by prefectures increased slightly from 94,500 to 2009 to 116,500 in 2020, whereas the number of services provided by municipalities increased from 10,400 to 2015 to 55,700 in 2020.

## Discussion

Between 2015 and 2020 in Japan, through two policy changes, provision of services by municipalities and the involvement of multiple health professions, there has been increased use of the three services related to swallowing/feeding difficulty. The first policy change led to a 1.8-fold increase in the use of Enhanced Oral Function. The second policy change to Maintenance of Oral Ingestion surged immediately after the 2016 but has remained flat since 2016. The Transition to Oral Ingestion decreased gradually from 2016 onwards. Following the two policy changes in 2015, Enhanced Oral Function and Maintenance of Oral Ingestion came to be used more frequently by older adults with slight disabilities.

The increased use of Enhanced Oral Function is a good example of a new style of service provision in which municipalities were given the authority to provide the service. The provision in the context of relationships with familiar faces in the community where older adults live may have increased participation. Preventive activities are likely to be offered to older adults who have not yet experienced severe swallowing/feeding difficulty and are at low risk of aspiration. In other words, preventive activities may be feasible without the need for highly specialized skills. We believe that preventive activities may be feasible even without advanced specialized skills. Even in countries without LTC insurance systems, training facilitators who can provide community-based prevention related to oral intake may reduce the incidence of malnutrition. Continued evaluation of its efficacy is needed in the future because active participation in primary prevention is a significant factor in preventing future development of malnutrition and respiratory disease [1, 20, 26]. If successful, these services are expected to extend the healthy life expectancy of older adults and reduce the burden of national medical expenditures.

Although Enhanced Oral Function has strengthened the aspect of primary prevention, this service was only used by a small portion of daycare service users at 6,800 times per 100,000 in-home service users. Moreover, usage by independent older adults has not expanded. Independent older adults may experience psychological barriers to receiving the same training in the same place as those with an LTC insurance certification. Training in a together setting may hurt their self-esteem, lessen their will to stay healthy, evoke images of a weak future and make them feel insecure.

The number of users of Transition to Oral Ingestion had been in decline, which was further accelerated by the Medical Fee revision. If this Medical Fee revision has indeed accelerated the decline in the inappropriate deployment of tube feedings, resulting in reduced use in Transition to Oral Ingestion, we can conclude that the use of both medical and LTC services has been appropriate.

Even though Enhanced Oral Function and Maintenance of Oral Ingestion services are on the rise, given the high prevalence of dysphagia among the older population, these services are provided to only a small fraction of those with hidden swallowing/feeding difficulty [7–11]. Despite several policy changes in LTC insurance for swallowing/feeding difficulty, underuse of the oral ingestion service may be attributed to two reasons. First, human resource development may be insufficient for the multidisciplinary collaboration that was the aim of the 2015 policy change to Maintenance of Oral Ingestion. Recommendations for multidisciplinary involvement in swallowing/feeding difficulty have been noted in many previous studies [16, 19, 20]. Clave et al. argued that specialized swallowing therapists who combine knowledge from multiple medical and surgical disciplines are needed [16]. Although the Japanese Society of Swallowing and Rehabilitation conducts the certification of swallowing therapists, the total number of physicians, dentists, dental hygienists, nurses, speech-language-hearing therapists, physical therapists, occupational therapists, and other professionals certified by the Society was only 3,349 in 2021 [27]. Furthermore, there were 1,042 highly skilled nurses with certification in dysphagia in 2021, of whom only 12 worked in LTC facilities, and there were only two medical education courses at two universities that offered accredited training [28]. Second, physicians and dentists now participate in implementing services for swallowing/feeding difficulty, but their numbers are fewer than the potential need in LTC facilities. A previous study that examined LTC facilities with cooperating dentists (n = 367) and the number of these cooperating dentists (n = 628) revealed a gap between the needs of LTC facilities and the implementation by dentists [29]. Regarding evaluation of swallowing, which is the basis for Maintenance of Oral Ingestion, 40.7% of LTC facilities desired this evaluation; however, only 21.0% of these facilities received this evaluation by cooperating dentists. Similarly, 30.9% desired and 18.9% implemented dentist participation in dietary observation, and 32.0% desired and 13.4% implemented participation in oral and nutritional conferences [29]. Another survey on Enhanced Oral Function reported that 24% of 1,062 daycare facilities that did not implement this service cited “lack of dentists willing to cooperate” as the reason [30].

One way to expand human resources would be to include in the system the promotion of facility visits, including daycare visits, by otolaryngologists who have sufficient expertise in feeding and swallowing. Although swallowing/feeding difficulty services are provided by staff with highly specialized skills, a basic education program should also be developed for facility staff, such as care support specialists and kitchen staff. The status of staff education in LTC facilities under the discretion of dentists and physicians is unknown. Although the LTC insurance policy change in 2021 requires new roles for registered dietitians in LTC facilities, including participation in nutritional dietary observation, a previous study has reported inadequate dysphagia management training in facility and kitchen staff [31]. In addition to feeding and swallowing rehabilitation, compensatory therapies to adjust the texture and moisture volume of food as well as maintenance of the eating posture are important in the treatment of dysphagia [32]. It is inappropriate to change the shape of foodstuffs to an excessive degree; however, there are many strategies that can be utilized before this is necessary [33]. Appropriate meal preparation based on meticulous dietary observation will enable tailoring of meals according to swallowing/feeding difficulty. The staff of LTC facilities are at the forefront of preventing aspiration and malnutrition in daily dietary care. Further research is needed on barriers to use, including human resource development, to promote the greater use of these two services in the future.

The three services in this study are focused on oral intake for older adults with swallowing/feeding difficulty at various stages, from prevention to the withdrawal of tube feeding. Oral intake means more than simply “adequate nutrition and hydration.“ The practices of oral intake involve the core identity of an individual at the personal and social levels [34, 35] and are a symbolic act of family and patient relationships, especially at the end of life or during life-threatening illness [18, 36]. Malnutrition in older adults is a major social problem in Western countries that is also straining healthcare expenditures, and in the United Kingdom, the NHS has developed guidance [37] and further education [38] for healthcare providers and caregivers with the aim of “adequate nutrition and hydration”. However, the main objective is to achieve the goal of nutrition and hydration; oral intake is not its primary theme. Whether this gap in awareness of oral intake and malnutrition among older adults is due to the existence of a well-insured older adult healthcare system or to Japanese culture is unclear.

### Limitations

This study has several limitations. First, because this study was conducted with home-dwelling LTC insurance-certified older adults who use daycare facility services and with residents in LTC facilities, oral-related services received by home-dwelling LTC insurance-certified older adults who do not use daycare were not evaluated. The second limitation relates to the analysis of the administrative database. As sex and age are not publicly available, they could not be included in the present analysis. The same service name may have had different service implementations due to the LTC service name indices. In this study, data were extracted accounting for fundamental changes in Maintenance of Oral Ingestion in terms of content and service infrastructure before and after 2014. However, no data were available for this service from 2009 to 2014. Third, the health outcomes of the provided services were not considered because data regarding the number of times LTC services were used provide no information about what care was actually provided.

## Conclusions

Support services for swallowing/feeding difficulty in the Japanese LTC insurance system have shown an upwards trend through two main policy changes. Looking at the details of the increase in these three services, the provision of Enhanced Oral Function as primary prevention by municipalities has contributed to this increase. There has been a shift in the main users of Enhanced Oral Function and Maintenance of Oral Ingestion to older adults with milder physical/mental conditions.

### Electronic supplementary material

Below is the link to the electronic supplementary material.

Supplementary Material 1

## Data Availability

Data were derived from a source in the public domain (https://www.e-stat.go.jp/stat-search/files?page=1&toukei=00450049&tstat=000001123535, in Japanese only).

## Abbreviations

LTC: long-term care
IDC-11: International Classification of Diseases version 11
PEG: percutaneous endoscopic gastrostomy
PTEG: percutaneous trans-oesophageal gastro-tubing
